# The needs analysis of the marriage education program for Turkish Cypriot community: Development phase

**DOI:** 10.3389/fpsyg.2022.963305

**Published:** 2022-08-02

**Authors:** Nihal Salman, Kemal Akkan Batman, Yasemin Sorakin

**Affiliations:** ^1^Near East University, Nicosia, Cyprus; ^2^Atatürk Teacher Training Academy, Nicosia, Cyprus

**Keywords:** marriage, marriage education needs, marriage education program, society and culture, divorce–psychology

## Abstract

This research is a needs analysis to develop a marriage education program to be held in the TRNC. The aim of the research is to compare the views of married and divorced individuals about marriage education and to determine their needs for marriage education. For this purpose, it has been determined in which subjects they see themselves as sufficient or inadequate, in which subjects they are willing to participate in the training and in which subjects they are unwilling. In this study, embedded design, one of the mixed research methods in which qualitative and quantitative methods are used together, was used. The dominant dimension of this study is the qualitative research method, and the supporting dimension is the quantitative research method. In the qualitative aspect of the research, the sample group was studied with 16 married and divorced participants living in the TRNC. In the quantitative dimension, a total of 385 married or divorced participants were consulted using the sample calculation of unknown universe. The findings are as follows; In both quantitative and qualitative dimensions, the majority of the participants think that if marriage education is given, they can have a healthier and happier marriage. Married and divorced individuals, effective communication, ways of conflict resolution, respect for differences in understanding in raising children, the causes and consequences of cheating, the importance and functions of the family, expectations from marriage and spouse, the processes experienced by new couples, attitudes and behaviors that may adversely affect marriage, power balance and marital status. They stated that they wanted to receive training on adjustment, relations with families in marriage, marriage and business life, anger control, coping with violence and prevention, spending habits and its effects on marriage, effects of financial difficulties on marriage, sexual life and problems in marriage. A training program will be developed for these determined subjects and will be applied to 20 participants in a semi-experimental model through the TRNC Ministry of National Education. The marriage psycho-education program to be prepared will be developed on the basis of a cognitive behavioral approach.

## Introduction

As COVID-19 spread around the world, measures for isolation began to be taken in line with the national health systems used in various countries. In many countries, it is aimed to prevent the spread of infection to a large extent with the implementation of measures such as maintaining social distance and personal isolation at home. Flexibility such as working from home and alternating work has been introduced to the working style of the public and private sectors, distance education has been introduced at all education levels, meetings, events and activities have been postponed or canceled (TUBA (Turkish Academy of Sciences), 2020). These measures have changed the ordinary course of individual and social life, caused significant life differences and affected all members of the society in different ways (Akoglu and Karaaslan, 2020). Factors such as being closed at home, social isolation, economic difficulties, and fear of death have adversely affected mental health and human psychology. In a study conducted in the USA, it was stated that one out of every three citizens had symptoms of clinical depression and anxiety (Bruine de Bruin et al., 2020). This situation has caused great changes in family life (Bruine de Bruin et al., 2020).

Changes in families during the COVID-19 process have caused social changes. While the pandemic caused a global crisis in the macro plan with its threatening nature to humanity, it caused problems in marriages and on an individual basis in the micro plan. Strict measures such as the curfew taken to slow the spread of the pandemic and the uncertain aspects of the disease trigger anxiety in individuals (Mahase, 2020). The interruption of educational activities, which is one of the effects of the pandemic, and the decrease in the income of individuals, caused an increase in stress in parents. According to the study of Taubman-Ben-Ari et al. (2021), parents experienced higher levels of stress during the pandemic period than before the pandemic. In this process, fathers had to stay at home differently from their previous lives, interacted with children intensely and unaccustomedly, increased their stress levels and experienced psychological disorders (Mahase, 2020). It has been observed that individuals' search for meaning and marital satisfaction expectations are at a high level during the COVID-19 process. It was observed that as the marital satisfaction level increased, the stress of parenting decreased. Taubman-Ben-Ari et al. (2021) stated in their study that it is important to focus on the meaning of life and strengthen marital relations in order to cope with increasing parenting stress.

The economic and social changes resulting from the closures have forced families to deal with unexpected upheavals in their homes and work environments. This sudden change in work and school accessibility has changed the time families spend together and the responsibilities that parents manage. School and home environments are combined through virtual learning platforms. As a result of the changes experienced, children who go to school stay at home full-time, increasing the responsibilities of parents (Singletary et al., 2022).

It has been suggested that profound changes in daily family life caused by the pandemic may increase parental stress and family tension, leading to an increase in negative childhood experiences, including domestic violence, child abuse and neglect (Mahase, 2020). Lee et al. (2020) investigated the effects of the COVID-19 pandemic on Korean families. Changes in families in terms of family time and relationships, childcare, school and education, economic welfare and employment, inequality and discrimination were examined. Relational difficulties have been observed in families who lost their jobs due to the pandemic. It was observed that domestic violence and child abuse increased during this period. Especially in socioeconomically disadvantaged families living in small houses, conflicts have been observed intensely due to violations of personal space (Lee et al., 2020). Tackling the challenges brought by the pandemic has further revealed the importance of family education (Guetto et al., 2021).

Education makes individuals conscious about marriage and parenting. According to Bilen (1983), the ability of individuals to establish a successful marriage depends, first of all, on their education that prepares them for marriage. Thus, the divorce rate decreases and relationships can be established in a healthier way. According to Fritsch (1985), parents are first and foremost victims of their ignorance. No one teaches them what problems they will face in marriage, how to overcome them, when to compromise and how to keep mutual love alive. Every divorce is an indication of an inadequate conclusion of a learning process. There are two types of activities aimed at preventing divorces and creating happy families. The first is family counseling, and the other is marriage and family education. Family counseling activities are aimed at preventing divorces and solving problems. In preventive and improving activities aimed at helping the family to be established and maintained in a healthy way from the beginning, the main purpose is to prevent situations that cause divorce. Education gains importance in these activities (Guner, 2014).

It has been determined in the literature that young people view premarital education very positively and they want to participate in premarital education programs (Silliman et al., 1992; Duncan et al., 1996; Martin et al., 2003; Silliman and ve Schumn, 2004). There is a wide variety of programs on “Relationship and Marriage Education” in the United States (Ponzetti, 2016). Relationship and Marriage Education Programs can be found in mental health centers, hospitals, public aid offices, churches or universities, etc. are made available to the public, such as Training venues vary according to their programme, learning format and target audience. Marriage education programs generally include communication, conflict resolution skills and family economics (DeMaria, 2005). Marriage Education programs appeal to individuals (for example, young people, fathers, mothers), couples (for example, premarital, married) and many groups for families (Ponzetti, 2016).

According to the TRNC Courts Annual Report for 2020, the total number of marriages in 2020 is 951, while the total number of divorces is 841. Divorce rate according to marriages is 88.4%. Worldwide, marriage is becoming less and less popular, and divorces are on the rise. Every year, organizations such as the United Nations announce global divorce rates. According to the data, divorces have increased by 252% globally since 1960, with the world's highest divorce rate (87%) in Luxembourg (https://ungo.com.tr/2020/12/ulkelere-gore-bosanma-oralari/). Considering these statistics, if the TRNC could enter the world statistics as a recognized country, it would be the country with the highest divorce rate in the world. The healthiest way to prevent divorces is through marriage education (Silliman et al., 1992; Duncan et al., 1996; Martin et al., 2003; Silliman and ve Schumn, 2004; DeMaria, 2005; Sürerbiçer, 2008; Canel, 2012; Öztürk and Arkar, 2014; Ponzetti, 2016; Yusof et al., 2017).

### Importance of research

This research was conducted to determine the needs of individuals regarding marriage education for the marriage education program envisaged to be carried out in the TRNC. Education is the most important preventive and developing activity to help the family to be established and maintained in a healthy way from the beginning. The main purpose of marriage education is to prevent situations that lead to divorce. No research has been found to reveal the needs of marriage education in the TRNC. The importance of this study is that it helps to fill the gap with its contribution to the gap in this field by revealing the needs of married and divorced people regarding marriage education. In addition, the study is important because of its contribution to the creation of a marriage education program design in line with the needs that will arise in the research. In line with this purpose, the problem sentence of the research is “determining the marital education needs of married and divorced individuals.”

### Purpose of the research

The aim of the research is to make a needs analysis to develop a marriage education program. Marriage education needs of married and divorced individuals were determined. For this purpose, a comparison was made according to the variables of which subjects they see themselves as competent or inadequate, in which subjects they are willing to participate in education and in which subjects they are unwilling, married and divorced.

The questions sought to be answered in the research are as follows:

1- What are the views of married and divorced individuals about communication in marriage?
2- What are the views of married and divorced individuals about cheating in marriage?
3- What are the opinions of married and divorced individuals about readiness for marriage?
4- How do married and divorced individuals cope with stress in marriage?
5- What are the views of married and divorced individuals about violence in marriage?
6- What are the views of married and divorced individuals about the economy of marriage?
7- What are the views of married and divorced individuals about sexuality in marriage?
8- What are the views of married and divorced individuals about their marriage education needs?
9- What are the marriage education needs of adults?

## Method

### Model

This research is a mixed study conducted to determine and analyze the marital education needs of married and divorced individuals living in the TRNC. Mixed method refers to the collection and analysis of qualitative and quantitative data together (Creswell, 2017). Embedded design, one of the mixed research methods, was used in this study. According to the embedded mixed method, data are collected simultaneously during the research, but a data format plays a supporting role (Hunt, 2007). The dominant dimension of this study is the qualitative research method, and the supporting dimension is the quantitative research method.

### Working group

The inclusion criteria of the participants in the study were to be married or divorced. People who met these criteria participated in the study. Gender has also been treated sensitively, and equality has been tried to be achieved.

#### Qualitative dimension of the research

Since the purpose of the embedded design study is to discover the conceptual structure related to the social processes of the phenomenon, the process of forming the working group is initiated by the researcher by observing the participants and interviewing meaningful individuals. In order to explore different dimensions of social processes, people with different experiences about the phenomenon are included in the study group. Analysis and collection of data is carried out simultaneously. The researcher continues to include people in the study group until saturation is reached. For this reason, the size of the study group varies between 10 and 30 people in embedded design studies (Starks and Trinidad, 2007). When the researchers encountered repetitions in the answers given by the participants, they realized that the data reached saturation and detailed the data instead of diversifying it. The sample group of this research consists of 16 married and divorced individuals living in the TRNC. Of the 16 participants, 8 were married and 8 were divorced. Of the married and divorced individuals, 4 are women and 4 are men. Selected participants continued the research until the end.

#### Quantitative dimension of the research

In the quantitative dimension of the research, it includes all married or divorced individuals living in the TRNC. Since it is not possible to reach the number of married and divorced individuals living in the TRNC, 384 people were determined in the study with a sampling formula of unknown universe, with a 95% confidence level and a 5% sampling error (Baştürk and Taütepe, 2013). The research was completed with 385 participants reached online. Two hundred thirteen participants are men and 172 participants are women. The number of divorced participants is 172, and the number of married participants is 213.

### Data collection agents

#### Interview form

In this study, a semi-structured interview form prepared by the researchers was used as a data collection tool. While preparing the interview form, first of all, a literature review was conducted to determine the dimensions related to marriage education (Sürerbiçer, 2008; Hawkins and Fackrell, 2010; Gur and Kurt, 2011; Canel, 2012; Hughes et al., 2012; Holland and De Valk, 2013; Öztürk and Arkar, 2014; Ponzetti, 2016; Mehrolhassani et al., 2018). As a result of the literature review, it has been determined that marriage education consists of communication, infidelity, readiness for marriage and choosing the right spouse, coping with stress, violence in marriage, economy and sexuality. Then, a total of 13 questions were prepared by interviewing experts (2 Clinical Psychologist and Family Therapist, 1 Curriculum and Instruction and 1 Turkish Language and Literature) to prepare the interview form. The 13 questions determined were sent to 2 clinical psychologists and family therapists for their opinions, and the final form consisted of a TOTAL of 8 questions. The form was named as “Marriage Needs Determination of Married and Divorced Individuals.”

#### Survey

While preparing the questionnaire, a total of 25 questions were prepared by making use of the literature reached while the above interview form was being developed. The questions were sent to the experts for content validity and their opinions were taken. In line with the opinions of the experts, the questionnaire included 6 items related to demographic characteristics and 20 items to determine the need for marriage education. The survey consisted of 8 main titles. The answers to the questions were formed as “Yes” or “No.” The questionnaire was named as “Marriage Education Needs Determination Questionnaire.”

### Analysis of data

In the qualitative aspect of the study, the researchers determined and tabulated the frequencies of the codes suitable for the determined themes. Coding is the classification of data according to concepts, titles and themes (Özdemir, 2010). Coding in qualitative research occurs in three ways. These; It is “coding according to predetermined concepts,” “coding according to concepts extracted from data,” “coding done within a general framework.” The coding used in this research is the coding made according to the concepts extracted from the data. In this type of coding process, data is evaluated and codes are created from similar answers (Yildirim and Simşek, 2011). Expert opinion and participant confirmation were made for the reliability and validity of the Interview Form. Asking participants whether the study findings accurately reflect their thoughts is called member checking (Erlandson et al., 1993). At the end of the data collection, the researchers summarized the data they collected and asked the participants to express their thoughts on the accuracy of these. In addition, the participants had the opportunity to add any perceptions or experiences they wanted to add. All of the participants stated that the results reflect their own views. Each stage of this research is explained in detail. In addition, transferring the participants' own statements as they are is a feature that increases the reliability and validity of the research. The audio recordings taken as a result of the interviews obtained within the scope of the research were transcribed verbatim at the end of the interviews and a data set of approximately 60 pages was obtained.

In order to determine the reliability of the answers given to the marriage education needs questionnaire, the Cronbach Alpha test, which is an internal consistency test, was applied and the alpha coefficient was found to be 0.982. The fact that the alpha coefficient is above 0.70 indicates that the measurement tool is reliable (Büyüköztürk, 2011). Expert opinion was taken for the content validity of the questionnaire. In the quantitative dimension of the research, the quantitative data obtained for statistical analysis in SPSS 20.0 program; were evaluated as descriptive statistical methods (number, percentile).

### Research process

Ethics Committee Permission was approved by the ethics committee on 04.11.2021 as the project numbered NEU/EB/2021/738.

In the qualitative aspect of the research, basic information about the purpose and method of the research was given to the participants, and written and verbal informed consent was obtained from each participant. It has been stated that care will be taken at the point of sharing self-explanatory information, and a code will be given to each participant to ensure anonymity. E1, E2, etc. to participants who are married. B1, B2 etc. to the divorced participants. It is preferred to give a code in the form of. In the research, data were collected through in-depth interviews. Harland (2014) states that the best way for individuals to obtain information about their own lives is to ask them questions in a way that allows them to express themselves using their own expressions, and this can be achieved through in-depth interviews. In the semi-structured interview form, introductory information was first asked from the participants. Then, open-ended questions were asked about communication in marriage, infidelity, readiness for marriage, coping with stress, violence in marriage, economy in marriage, sexuality and marriage education needs. During the in-depth interviews with the participants, the consent of the participants was obtained for the use of a voice recorder.

In the quantitative aspect of the research, the survey questions were prepared online due to the inconvenience of one-on-one interviews during the pandemic process. In the research, a survey form was sent to randomly selected married and divorced individuals online between 01 November 2021 and 30 May 2022 using Google Forms method. Before starting the research, a pilot study was conducted with 10 married or divorced individuals to access and understand the data forms.

## Findings

### Findings regarding the qualitative dimension of the research

As seen in Table 1, in the questions about communication addressed to a total of 16 individuals, 8 married and 8 divorced, who participated in the study, all participants answered “Communication is the most important thing.” they replied. On the theme of the effect of problems with relatives and friends on marriage, 4 married and 5 divorced participants say negative effects, 3 married and 3 divorced participants do not; 1 married participant said that we try not to reflect it. When the participants were asked about their methods of solving the problem with their spouse, 8 divorced individuals replied, “We couldn't solve it.” Then, 4 married individuals said, “We solve it by getting away from the discussion and then talking.” 3 married individuals gave the answer “trying to understand each other...,” 1 married individual replied “silent.” When the participants were asked about their methods of resolving differences of opinion in decisions about children, 6 married and 2 divorced individuals said, “We make joint decisions by talking.” Then, there are 1 married and 4 divorced individuals who say “We argue a lot, we can't get along”; 1 married and 2 divorced individuals said, “Usually, he leaves the decision to me.” he said.

**Table 1:** Opinions on the need for marriage education.

**Themes**	**Codes**	**Married (f)**	**Divorced (f)**
The importance of communication in marriage	1. Communication is the most important thing.	8	8
The effect of the problem with relatives and friends on marriage	1. Adverse effects 2. Does not affect at all 3. We try not to reflect.	4 3 1	5 3 0
Methods of solving the problem with the spouse	1. We couldn't solve it. 2. We solve it by walking away during the discussion and then talking. 3. By trying to understand each other. 4. Silently.	0 4 3 1	8 0 0 0
Methods of resolving disagreements in decisions about the child	1. We make a joint decision by talking. 2. We argue a lot. 3. He usually leaves the decision to me.	6 1 1	2 4 2
Opinions about cheating	1. Is disrespectful. 2. It's the end of t trust. 3. It is the reason for the marriage to end. 4. It is an indication that the sexual need is not met in marriage.	3 2 2 1	2 3 2 0
Have you been cheated on?	1. No. 2. Yes.	8 0	7 1
Did you cheat?	1. No.	8	8
Methods of coping with cheating	1. I lost my self-confidence. 2. I have never experienced it. 3. I was unresponsive at first, divorced after many times	0 8 0	1 6 1
Psychological readiness for marriage	1. Yes. 2. No.	3 5	1 7
Social readiness for marriage	1. Yes.2. No.	7 1	4 4
Economic readiness for marriage	1. Yes.2. No.	6 2	4 4
Do you believe you are marrying the right spouse?	1. Yes.2. No.	7 1	0 8
Ways to overcome stress	1. I withdraw into myself, I dissolve within myself. 2. I solve it by arguing. 3. I stay away from the environment. 4. I find it difficult to overcome.	1 3 4 0	2 2 2 2
The state of controlling anger	1. I usually do. 2. I can't.	7 1	5 3
Exposure to violence	1. Yes.2. No.	0 8	2 6
Violence use status	1. No 2. Yes.	8 0	7 1
Definition of violence	1. The weakness of masculinity. 2. Inability to express oneself; is weakness. 3. Animalism. 4. On-site required.	3 4 1 0	2 5 0 1
Compatibility with the spouse when balancing the family budget	1. We are compatible. 2. We are incompatible. 3. We are not very compatible, somehow we get along.	6 1 1	1 7 0
Methods of ensuring compliance	1. By talking, we make a joint decision. 2. Everyone is responsible for their own budget 3. It was our biggest reason for discussion.	5 3 0	1 3 4
Situations in which economic problems affect marriage	1. It affects a lot. 2. It does not affect much. 3. It does not affect our marriage, but affects our purchasing power.	6 1 1	7 1 0
Having sexual problems with your partner	1. No. 2. Yes. 3. We lived in the first years of marriage, then we overcame the problems.	5 2 1	3 4 1
Definition of sexuality in marriage	1. It is one of the cornerstones of marriage.	8	8
Sexual compatibility with the spouse	1. We are compatible. 2. We are not compatible. 3. We are not very compatible, we manage.	4 1 3	1 6 1
Would you have a healthier and happier marriage if you were given training on the above subjects?	1. Yes, it would. 2. No, it would not. 3. It would be nice if there was training on some subjects.	5 1 2	6 1 1
On which subjects would you have a happier and healthier marriage? *	1. Correct communication method. 2. Problem solving skills. 3. Sexuality. 4. Choosing the right mate. 5. To be a parent.	7 8 3 2 6	8 8 3 5 4

* Participants gave more than one code.

As can be seen from Table 1, the participants said that communication is important in marriage. Detailed answers to this question are below:

“*..... It is very important. It causes problems to be solved. When I have problems with my relatives and friends, it does not affect my marriage, I do not reflect it. When I have a problem with my partner, I walk away from the scene, and when we're both calm, we solve the problem by talking. We try to come to a consensus on our disagreements about our children, give his ideas and mine. We choose whichever is best.” E2*

“*……if there is no communication, that marriage will end anyway. Problems with relatives did not affect my marriage at all. Because my relationship with my relatives ended in 1984. I am not related to any of my relatives. So it wouldn't affect my marriage. Despite this, I got divorced, so relatives do not cause divorce. We could not solve the problems we were experiencing. After constant arguments, my marriage ended. It would be what I said in the decisions about the child.” B7*

As can be seen in Table 1, the individuals participating in the research have the theme of “Opinions on Cheating in Marriage,” 3 married and 2 divorced individuals “It is disrespectful.”; 2 married and 3 divorced individuals “It is the end of the trust”; 2 married, 2 divorced individuals, “It is the reason for the end of the marriage.”; 1 married individual said, “It is an indication that sexual needs are not met in marriage.” “Have you been cheated on in your marriage?” To the question, 8 married and 7 divorced said “no,” while 1 divorced said “yes.” “Did you cheat?” All participants said no to the question. In the theme of “Coping with Cheating,” 1 divorced individual said “I lost my self-confidence.”; While 8 married, 6 divorced said I have never lived, 1 divorced individual said, “I was unresponsive at first, I got divorced many times.” he said.

The opinions of the participants about cheating are given below:

“*……… must not be deceived. I think people's respect for marriage and a sense of loyalty are very important. Cheating people will cause great wounds to the marriage, and it will prevent that marriage from working in a healthy way. Cheating is not just a physical activity. I think cheating is something that a person can do in his brain as well. Desiring for another person in situations where one is not self-sufficient or insufficient is to want. Dangerous feelings are situations that should not be in marriage. I've never cheated, I don't think I've been cheated on.” E8*

“*…….. Let me tell you, we are human, sometimes we squint, how can I explain small deceptions, he made a mistake once, he accepted it, I can forgive him. But if it goes for a long process or cheats with different people, I can't accept it. Cheating is the shifting of both your mind and heart out of the family. I have been cheated on several times. Well, at first, I left it to the process. That he would see the mistake and return, and that's what happened. But once he did, he did the second, he did the third, which caused violent arguments so we got divorced. I didn't cheat. Being cheated on is very hurtful. It was a difficult process. At first, I closed myself off from everything. I was very sorry. But over time, I did not get any support and help by inculcating myself. But I wish I had. I believed that I would go through this process properly. That's how I tried to deal with it.” B3*

When the views on readiness for marriage are evaluated in Table 1, 3 married and 1 divorced individuals stated “I was psychologically ready.”; 5 married, 7 divorced individuals “No, I was not ready.” he said. “I was socially ready.” While 5 married and 4 divorced individuals said “yes” to the theme, 3 married and 4 divorced individuals said “no.” “I was economically prepared.” 6 married and 4 divorced said “yes” to the theme, while 2 married and 4 divorced said “No, I wasn't.” “Do you believe you married the right wife?” To the question, 7 married individuals said “yes,” 1 married and 8 divorced individuals said “no.”

“*……When I got married, I was not ready for marriage psychologically (age, taking responsibility, maturity, etc.), I was ready for marriage socially (completing education, stepping into the profession, etc.), I was ready economically (house, car, salary, etc.). I don't believe I married the right wife. I believed it at first. Then we realized that we are very opposite characters. Maybe that pushed him out. Maybe that's why he cheated.” B4*

“*……I wasn't ready for marriage psychologically (age, taking responsibility, maturity, etc.) when I got married, let me tell you, I was ready for marriage socially (completing education, starting a career, etc.), economically (house, car, salary, etc.) I was not ready. It's never ending. I do not believe that I married the right wife.” E7*

When the views on coping with stress are evaluated in Table 1, 1 married and 2 divorced individuals said, “I shut myself in, I solve it inside myself.”; 3 married, 2 divorced individuals, “I will discuss and solve it”; 4 married, 2 divorced individuals “I stay away from the environment.”; 2 divorced individuals: “I find it difficult to get over.” replied in the form. To the question of controlling anger, 7 married and 5 divorced individuals answered, “I usually do.” Then, 1 married and 3 divorced individuals said “I can't.”

“*……….. There is nothing without stress. This includes marriage. You can live in many material and spiritual matters. Material things come and go. In spiritual ones, people want psychological support. Sometimes he overcomes it by talking to a friend, sometimes he overcomes it by closing himself in, somehow people overcome such feelings with the urge to continue living. I cannot control my anger.” E1*

“*………. I went through a lot of stress in marriage. My marriage passed without money. I did not have any financial support. Live alone in the heart of Istanbul. Have no one, no supporter, do you beg for money on the street, no job, no power, and go to university. I used to live in Istanbul, I would go to Edirne for university. I had a hard time overcoming these stresses I was going through. I couldn't control my anger.” B7*

As seen in Table 1, when the “opinions about violence in marriage” of the individuals participating in the research were evaluated, 2 divorced individuals said “yes” to “exposure to violence,” while 8 married and 6 divorced individuals said “no.” As for the “infliction of violence,” 8 married and 7 divorced individuals said “no,” while 1 divorced individual said “Yes.” he said. To the question of “Definition of violence,” 3 married and 2 divorced individuals said “weakness of masculinity,” 4 married and 5 divorced individuals answered “Not being able to express oneself,” 1 married individual is “Animal,” 1 divorced individual is “Necessary in place.” he said.

“*…… I was not exposed to violence from my wife, nor did I show it. Violence is the end of marriage. If you're going to be violent toward your wife, it means you don't want her. The one who moves to finish gets fed up with him and says enough is enough, he packs his bag and leaves or you go. For me, using violence shows the weakness of masculinity.” E6*

“*……. Men are not likely to be exposed to violence. I have abused my wife several times. I got even more enraged when I beat him. Because he didn't respond. When I beat my wife, I realized that violence is not the solution. Because I was even more enraged. Violence is necessary on the ground. Not on everything though. Violence is necessary when appropriate.” B6*

As seen in Table 1, the individuals participating in the research were asked “We are compatible,” “We are incompatible,” “We are not very compatible, we get along somehow.” have given their answers. Six married and 1 divorced participant, “We are compatible.” while 1 married and 7 divorced “We are mismatched.”; 1 married person said, “We are not very compatible, we get along somehow.” they said. When the participant individuals were asked about “methods of ensuring adaptation,” 5 married and 1 divorced individuals replied, “We make a joint decision by talking”; 3 married and 3 divorced individuals said “Everyone is responsible for their own budget.” he said. “It was our biggest argument.” The one who says is 1 divorced person. In response to the question of “Economic problems affect marriage,” “It affects a lot.” said 6 married and 7 divorced; “Doesn't affect much.” Those who say, 1 married and 1 divorced; “It doesn't affect our marriage, it affects our purchasing power.” Those who say are 1 married individual.

“*……. My wife and I are in harmony while balancing the family budget. We have joint account. We do this by talking to each other. Economic problems do not affect our marriage, but rather affect our purchasing power. We only delay getting our wants and needs.” E5*

“*……. While balancing the family budget, my wife and I were not in harmony. We were mostly penniless. Economic problems affect marriage very negatively, of course, it brings peace.” B5*

As can be seen from the table, when asked to balance the family budget, it was found that married people were more in harmony with their spouses than divorced individuals.

In Table 1, when the individuals participating in the research were asked about their “experiencing sexual problems,” 5 married and 3 divorced people said “No,” while 2 married and 4 divorced people said “Yes.” “We lived through the early years of marriage, then overcame problems.” and only 1 married and 1 divorced individual; When asked “definition of sexuality in marriage,” all respondents replied, “It is one of the cornerstones of marriage.” they said. “When asked about the status of sexual compatibility with the spouse, 4 married and 1 divorced individual replied, “We are compatible.”; 1 married and 6 divorced individuals say “We are not compatible.” he said. “We are not very compatible, we manage.” Those who say are 3 married and 1 divorced individual.

“*………. Sounds like we're having sexual problems in our marriage these days. It's clear that I'm going into andropause. And I'm laughing through it. I get through with a lot of laughs. I'm not hot like folks. Everything will come to pass. I think I am compatible with my wife. Not at this time though. Sex is the most important thing to me.” E6*

“*….. We may encounter problems of sexual origin in marriage. Sex, of course, means a lot. Since human beings have impulses, since such feelings must be experienced, sexuality should be experienced as a source of happiness, not a problem. It may be the thing my wife is most attuned to. I think men have more impulses than women.”*

“*…….. If there was marriage education, people would be more conscious. She continues her marriage without making any mistakes. If there was such an education, he could find answers to the questions in his mind, at least when he thought about what the problems might be. If there was a marriage education, I think it would be much better to know what marriage is like, not just a dream, but to know how to behave in terms of material and moral expectations, expectations of the spouse, sexual expectations, responsibilities, children, and how to behave in anger and arguments.” E3*

As seen in Table 1, the individuals participating in the research asked, “Would you have a healthier and happier marriage if you were given training on the above subjects?” When asked the question, 5 married and 6 divorced people replied, “Yes it would be.” While 1 married and 1 divorced person answered, “No, it would not be.”; 2 married and 1 divorced person said, “It would be nice if there was training on some subjects.” they said. Accordingly, 87.5% of the participants stated that they needed marriage education to the questions sought in the sub-objectives. The participants were asked, “What subjects would you have had a healthier and happier marriage?” When asked, 3 married and 4 divorced said “the right method of communication”; 7 married and 8 divorced people who said “correct communication method”; 8 married and 8 divorced; 3 married and 3 divorced saying “sexuality”; 2 married and 5 divorced saying “choosing the right spouse”; Those who say “don't be a parent” are 6 married and 4 divorced people.

“*………. I definitely believe that people should be informed before getting married. For example, if I received such an education, I am an aware person, if even an adult and a person around me sat down and talked to me, do not hurry, there are many years ahead, so continue your relationship, mature together. Look a little more if he had said to each other that way. Or marriage means this, there are these, are you ready, if he had talked to me, maybe even he would have helped me a lot. It is very important to receive training on relationships and communication. Marriage is a very important decision. For this reason, we need to question thoroughly what we want and why. It would be really good if pre-marriage trainings were made to raise awareness on this subject.” B4*

“*……….. I believe that if the training had been given, it would certainly have contributed, if not completely. You know, I believe that when married people have problems with this issue, there should be more authorities they can apply to, and the society should be made more aware of this issue and it should be made known that there are such opportunities. I think communication, going to a specialist about how we can solve mutual problems by talking, and getting information on this subject, I think, would have saved many marriages or would have been a factor in ending them more smoothly.” E2*

When the question was asked to the married and divorced participants, in which field they would have had a healthier and happier marriage, they answered the right method of communication, problem-solving skills, sexuality, choosing the right spouse, and being a parent. Almost all of the participants stated that it is necessary to receive training on correct communication method and problem solving skills. Divorced participants in choosing the right spouse think that more education is needed than married participants.

### Findings regarding the quantitative dimension of the study

When Table 2 is examined, 85.45% of the individuals included in the study answered yes to the opinion that marital education is effective in establishing and maintaining healthier marriages, and 2.86% answered no to the opinion that marital education is effective in establishing and maintaining healthier marriages. It was determined that 11.69% of them gave the answer perhaps to the opinion that marriage education is effective in establishing and maintaining healthier marriages. The education of 18.18% of the participants helps to make healthier decisions in choosing a spouse, 8.82% of them helps to make a more conscious decision to marry, 20.32% of them helps in solving the problems experienced or may be experienced in marriage, 12% It was determined that 0.83 of them found education helpful in being more successful parents, and 38.77% of them found education useful because it helps to lead a happier married life.

**Table 2:** Opinions of the participants on marriage education.

	**Dear (*n*)**	**Percent (%)**
**Marriage education in establishing healthier marriages** **opinion on the effectiveness of**		
Yes	329	85.45
No	11	2.86
Maybe	45	11.69
**Educational benefits**		
It helps to make healthier decisions in choosing a spouse.	68	18.18
It helps to make a more conscious decision to marry.	33	8.82
Helps in solving problems experienced or to be experienced in marriage	76	20.32
It helps to become more successful parents.	48	12.83
It helps to have a happier married life.	145	38.77
All	4	1.07

When Table 3 is examined, “Do you think healthy communication is important in marriage?” 83.64% answered yes and 16.36% answered no to the question, “Do you (did you) have communication problems with your spouse or ex-spouse?” to the question “If you have a child, do you have problems communicating with your child?” 40.78% of them answered yes to the question, 59.22% answered no. To the question, 52.73% answered yes and 47.27% answered no. 52.47% answered yes and 47.53% answered no to the question, “Do you think you would be more successful in solving communication problems in your family if you received effective communication training?” 75.58% of the respondents answered yes and 24.42% answered no to the question, “If it is given about conflict resolution methods; would you (were) more successful in overcoming conflicts in your marriage?” 75.58% of them answered yes and 24.42% answered no to the question, “If education is given about the reflections of the differences in understanding about raising children on marriage; would it have a positive effect on your marriage (did it)?” It was seen that 73.51% of the respondents answered yes and 26.49% answered no to the question. Regarding the subject of infidelity, the participants were asked, “Do you think every infidelity in marriage should result in divorce?” 58.70% answered yes and 41.30% answered no to the question, “Do you think it would be easier to cope with what happened as a result of cheating if training was given?” 63.12% of the respondents answered yes and 36.88% answered no to the question, “Would you feel guilty if you cheated on your spouse (would you)?” 70.65% answered yes and 29.35% answered no to the question, “Do you think cheating can be prevented if training is given about the causes and consequences of cheating?” It was seen that 60.26% answered yes to the question, and 39.74% answered no. On the subject of readiness for marriage and choosing the right spouse of the people participating in the research, “Will it contribute positively to marriage if training is given about the importance and functions of the family?” 77.14% of them answered yes and 22.86% of them answered no to the question, “Will it contribute positively to marriage if training is provided on the reflection of expectations from marriage and spouse on the marriage union?” 75.84% answered yes, 24.16% answered no to the question, “If it is given about the importance of getting to know each other, will it contribute positively to marriage?” 76.36% of them answered yes and 23.64% answered no to the question, “If it is given about the problems and solutions during the preparation for marriage, will it have a positive contribution to the marriage?” 77.66% of them answered yes and 22.34% answered no to the question, “If it is given about the processes that the newly married couples go through, will it have a positive contribution to the marriage?” 77.40% answered yes and 22.60% answered no to the question, “If it is given about habits, attitudes and behaviors that may negatively affect marriage, will it contribute positively to marriage?” 76.10% answered yes, 23.90% answered no to the question, “If it is given about power, balance and harmony in marriage, will it contribute positively to marriage?” 77.40% answered yes and 22.60% answered no to the question, “If it is given about relations with families in marriage, will it contribute positively to marriage?” 77.81% of them answered yes, 25.19% answered no to the question and “If it is given about marriage and business life, will it have a positive contribution to marriage?” It was seen that 74.81% of the respondents answered yes to the question, and 25.19% answered no.

**Table 3:** Distribution of respondents' responses to the marriage education needs questionnaire.

		**Yes**		**No**	
		***N***	**%**	***n***	**%**
Communication	Do you think healthy communication is important in marriage?	322	83.64	63	16.36
	Do you (did you) have communication problems with your spouse or ex-spouse?	207	53.77	178	46.23
	If you have children, do you have problems communicating with your child?	157	40.78	228	59.22
	Did (were) any problems or problems with your relatives affecting your relationship with your spouse or ex-spouse?	203	52.73	182	47.27
	Do you (did you) have problems with your spouse or ex-spouse because of disagreement when making decisions about your child?	202	52.47	183	47.53
	Do you think if you receive effective communication training, will you be more successful in solving communication problems in your family?	291	75.58	94	24.42
	If given about conflict resolution methods; would you (were) more successful in overcoming conflicts in your marriage?	291	75.58	94	24.42
	If education is given about the reflections of the differences in understanding about raising children on marriage; would it have a positive effect on your marriage?	283	73.51	102	26.49
Cheat	Do you think every cheating in marriage should result in divorce?	226	58.70	159	41.30
	Do you think it would be easier to cope with what happened as a result of cheating, if education is given?	243	63.12	142	36.88
	Would you feel guilty if you cheated on your spouse?	272	70.65	113	29.35
	Do you think cheating can be prevented if education is given about the causes and consequences of cheating?	232	60.26	153	39.74
Readiness for marriage and choosing the right spouse	If education is given on the importance and functions of the family, will it contribute positively to marriage?	297	77.14	88	22.86
	If training is given on the reflections of expectations from marriage and spouse on the marriage union:	292	75.84	93	24.16
	Given the importance of getting to know each other for couples getting married:	294	76.36	91	23.64
	If given about the problems and solutions during the preparation for marriage:	299	77.66	86	22.34
	If given about the processes experienced by newly married couples:	298	77.40	87	22.60
	If given about habits, attitudes and behaviors that may negatively affect marriage:	293	76.10	92	23.90
	If given about power, balance and harmony in marriage:	298	77.40	87	22.60
	If given regarding relations with families in marriage:	288	74.81	97	25.19
	If given about marriage and business life:	288	74.81	97	25.19

As you see Table 4, regarding the issue of coping with stress of the individuals included in the research, “Do you reflect your anger outside?” 47.79% answered yes and 52.21% answered no to the question “Can you control your anger?” To the question, 67.27% of them answered yes and 32.73% answered no, “Do you get extremely angry when you conflict with your spouse or ex-spouse?” To the question, 50.65% answered yes and 49.35% answered no. It was seen that 64.68% answered yes to the question, and 35.32% answered no. Regarding the issue of violence in marriage, the participants were asked, “Do you think violence affects marriage negatively?” 75.84% of them answered yes and 24.16% answered no to the question, “Do you think violence is an effective method in communication with both the spouse and the child?” 42.08% answered yes and 57.92% answered no to the question “Have you been exposed to violence from your spouse in marriage?” 33.77% of them answered yes, 66.23% answered no to the question, “Did you show violence to your spouse?” 29.61% answered yes and 70.39% answered no to the question, “Do you think if training on coping with violence and prevention is given, will it affect marriage positively?” It was seen that 71.43% answered yes to the question, and 28.57% answered no. Regarding the subject of economy of the individuals within the scope of the research, “Are you (were) in harmony with your spouse or ex-spouse while balancing the family budget?” 68.05% of them answered yes and 31.95% of them answered no to the question, “Did the economic problems affect your marriage (did it)?” 61.04% of them answered yes and 38.96% of them answered no to the question, “If education is given on spending habits and their effects on marriage; Will it contribute positively to the marriage?” 70.65% answered yes and 29.35% answered no to the question, “If it is given about the effects of financial difficulties on marriage; Will it contribute positively to the marriage?” 70.13% of the respondents answered yes and 29.87% answered no to the question. 42.08% answered yes and 57.92% answered no to the question “Are you (were you) sexually compatible with your spouse or ex-spouse?” 62.34% of them answered yes and 37.66% of them answered no to the question, “If you were given training on sexual life and sexual problems in marriage, would you (would you) have a happier marriage?” It was determined that 65.45% of them answered yes and 34.55% answered no to the question.

**Table 4:** Distribution of respondents' responses to the marriage education needs questionnaire.

		**Yes**		**No**	
		***n***	**%**	***n***	**%**
Coping with stress	Do you project your anger outwardly?	184	47.79	201	52.21
	Can you control your anger?	259	67.27	126	32.73
	Do you (where you) extremely angry when you conflict with your spouse or ex-spouse?	195	50.65	190	49.35
	If you received training on anger management, do you think it would (was) affect your marriage or ex-marriage positively?	249	64.68	136	35.32
Violence in marriage	Do you think violence affects marriage negatively?	292	75.84	93	24.16
	Do you think violence is an effective method in communication with both the spouse and the child?	162	42.08	223	57.92
	Have you been subjected to violence from your spouse in your marriage?	130	33.77	255	66.23
	Or did you show?	114	29.61	271	70.39
	Do you think it will affect marriage positively if training is given on coping with violence and prevention?	275	71.43	110	28.57
Economy	Are you (were) in tune with your spouse or ex-spouse when balancing the family budget?	262	68.05	123	31.95
	Did (were) economic problems affecting your marriage?	235	61.04	150	38.96
	If education is given on spending habits and their effects on marriage; Will it have a positive effect on the marriage?	272	70.65	113	29.35
	If it is given about the effects of financial difficulties on marriage; Will it have a positive effect on the marriage?	270	70.13	115	29.87
Sexuality	Do you (did you) encounter sexual problems in your marriage?	162	42.08	223	57.92
	Are you (were) sexually compatible with your spouse or ex-spouse?	240	62.34	145	37.66
	Would you (would you) have a happier marriage if education was given on sexual life and sexual problems in marriage?	252	65.45	133	34.55

## Discussion

In this section, the findings related to the order of the research questions are discussed one by one in the light of the literature. With the pandemic, there has been an increase in divorce rates in our country as well as all over the world.

All of the married and divorced individuals participating in the research are of the opinion that communication is very important in marriage. All of the participants stated that training should be given on the correct communication method. Family support can be a positive support for parents and children to experience psychological problems or to overcome psychological problems due to communication problems in marriage. In the study of Calvano et al. (2021), it was determined that the COVID-19 epidemic revealed the importance of communication within the family. As schools, childcare centers and workplaces are closed; family members stayed in the house longer, which changed the usual way of communication. In the study of Calvano et al. (2021), it was revealed that communication within the family is an effective factor that protects family and members in difficult situations. The fact that Kanter et al. (2021) emphasized the necessity of providing relationship education in order to protect marriages and prevent divorces is in line with the results of this study. In this study, it was determined that there were no great differences in the opinions of married and divorced individuals about whether problems with relatives and friends affect marriage. More than half of the participants are of the opinion that problems with relatives and friends affect their marriage negatively. Marriage is not only the togetherness of men and women, but also a process in which mother-in-law, father-in-law, sister-in-law, in-law, and brother-in-law can be included in the marriage. Kinship relations can vary from society to society. The most important factor in these kinship relations is the mother-in-law and father-in-law. Kinship relations that vary between cultures create positive and negative effects on marriages (Evran, 2018; Zöhre et al., 2021). Married couples stated that they tend to move away during conflict. Divorced spouses, on the other hand, stated that they could not resolve their conflicts. Accordingly, it can be said that conflict resolution skills are as important as communication in healthy marriages.

In this study, it was determined that there was no great difference between the opinions of married and divorced individuals about the phenomenon of infidelity in marriage. The participants see cheating in marriage as disrespect, the end of trust and the reason for the end of the marriage. The majority of the participants stated that they did not cheat on their spouses. Only one divorced participant stated that he cheated on his wife. When asked how you coped with being cheated on, a divorced person said that I lost my self-confidence, I accepted it at first, and then divorced many times. In the study of Salman et al. (2016) conducted in the TRNC, it was determined that there was a negative correlation between marital adjustment and conflict for both women and men, and it was determined that as the harmony increased, the conflict decreased. At the same time, it was determined that there was a positive correlation between the tendency to conflict and cheating in women and men, and as the tendency to conflict in marriage increased, the tendency to cheat also increased. In this sense, it is thought that trainings that increase harmony between spouses will reduce cheating as well as conflicts (Alaçam, 2020; Chalmers, 2020). The most basic way to increase harmony in marriage is marriage education.

In this study, most of the married and divorced individuals are of the opinion that they are not ready for marriage psychologically (age, maturity, taking responsibility, etc.) when they get married. Education is a big part of our life today. In the century we live in, we now know that people shape all their behaviors by learning. Marriage is a very important institution. It brings great responsibilities to the life of the individual, not only the individual, but also spouses and children are affected in every matter; even the whole life of children is affected (Sürerbiçer, 2008). It can be said that it would be a big mistake to enter a social group that has such great influences without being psychologically ready (age, maturity and taking responsibility, etc.). However, with education, it is possible to prepare individuals psychologically for what marriage is and what can be experienced, in short. Another code that draws attention in the research is the selection of the right spouse. While most of the married participants of the study believe that they are married to the right spouse, all of the divorced individuals are of the opinion that they do not marry the right spouse. In the study of Kashirskaya et al. (2015), psychological readiness for marriage affects marital relations and is an important factor in a happy marriage. The most effective way to be ready for marriage is through marriage education (Kashirskaya et al., 2015). The fact that most of the participants in this study stated that they were not ready for marriage and that especially all divorced individuals were of the opinion that they did not marry the right spouse, is in line with the findings of Kashirskaya et al. (2015).

The codes reached regarding the stress coping situations of the participants in marriage are as follows: “I shut myself in, I solve it inside myself,” “I solve it by arguing,” “I stay away from the environment,” “I find it difficult to overcome” In anger control, the answers were given as “I usually do” and “I can't.” It was observed that there was no great difference between married and divorced people in the views of the participants about the methods of overcoming stress. Individuals said that they overcame stress by withdrawing, arguing, and getting away from the environment. It can be said that divorced people are more unsuccessful in anger management. In the study of Hosgor (2013), it was revealed that there is a significant relationship between trait anger-anger expression styles and marital adjustment. It has been observed that individuals are more successful in anger and anger control as the harmony in marriage increases (Hosgor, 2013). In this sense, increasing harmony in marriage can also reduce violence. The harmony between spouses can be increased with marriage education (Hosgor, 2013). In this study, the finding that divorced individuals have less anger control than married individuals is in line with the results of Hosgor (2013).

In this study, the number of participants who said “I was exposed to violence and perpetrated violence” about violence in marriage was very few and they were also divorced. However, although violence is rare, it must be prevented. Sarica (2018) found that the perception of violence among students in the TRNC University Students' Perception of Violence Against Women is generally not very sharp compared to other countries. It is similar to the findings of this research.

In this study, it was determined that married participants related to marriage economy were more in harmony with their spouses than divorced individuals in balancing the family budget. Participants are of the opinion that economic problems in marriage affect marriage negatively. This shows that there is a need for economic education in marriage. In the study of Gurel (2016), it was determined that the economic difficulties experienced by the spouses decreased their marital quality through the mediation experienced by them and/or their spouses. The low marital quality of the spouse is largely due to his own burnout, and relatively due to the spouse's burnout. In summary, in the study of Gurel (2016), it was determined that economic difficulties in the family negatively affect the quality of marriage. This result is in parallel with the findings of this study.

In this study, while most of the married individuals stated that they did not have sexual problems with their spouses, most of the divorced individuals stated that they had problems. All of the participants defined sexuality in marriage as “one of the cornerstones of marriage.” While the majority of divorced individuals are of the opinion that they are not compatible with regard to sexual compatibility with their spouses, the majority of married individuals are of the opinion that they are compatible. Some of them are of the opinion that “we are not very compatible, we manage.” From this, it can be concluded that sexual compatibility is an important element in marriage. In the study of Nekoolaltak et al. (2019), it was determined that sexual compatibility increases the stability of marriage and partner relationships and reduces sexually transmitted diseases. Active participation in sexual relations, forgiveness and thoughtfulness, non-snoring, mutual respect, flexibility and sexual conversation with spouses, patience and development of sexual intercourse were identified as prominent features of sexually compatible couples (Nekoolaltak et al., 2019). In this sense, the harmony of the spouses is of great importance for a happy marriage. Relationships and sexuality of individuals who are not happily married are negatively affected. In order to ensure harmony in marriage, raising awareness of individuals and education for this is of great importance. In this study, it can be said that the inclusion of sexual issues in marriage education programs will contribute to the harmonious and sustainable marriages, considering the fact that the fact that divorced individuals have sexual problems will disrupt marital harmony.

The majority of the participants in this study think that if marriage education is given, they can have a healthier and happier marriage. Participants stated that they needed training on correct communication method, problem solving skills, sexuality, choosing the right spouse, and being a parent. Divorced participants need more training than married participants on the right choice of spouse training. In the study of Hamamci et al. (2011), participating university students mostly stated the need to receive training on developing bonding between couples, improving communication skills, learning to be a parent, and preventing divorce. In the study of Avci (2014), the subjects that university students most want to receive pre-marital education are, respectively; communication, conflict resolution, acceptance of differences, romance-sexuality and social support. In the study conducted by Snyder et al. (2010) with single and married individuals to determine their marriage education needs, it was found that most of the women needed training on domestic violence and cheating, while the men needed training on financial stability, communication and parenting skills. The results of the studies carried out to determine the need for marriage education show parallelism with the findings of this study. From this point of view, it can be said that adding the right communication method, problem solving skills, sexuality, choosing the right spouse, being a parent to the topics of the education programs to be prepared about marriage education will play a fundamental role in marital harmony and thus in maintaining a healthy marriage.

The qualitative and quantitative findings obtained from our study overlap. Responding to quantitative questions, the participants listed the subjects in marriage education as follows: effective communication, ways of conflict resolution, respect for differences in understanding in child rearing, the causes and consequences of cheating, the importance and functions of the family, expectations from marriage and spouse, processes experienced by new couples, negative marriage. They stated that they want to receive training on attitudes and behaviors that may affect marriage, power balance and harmony in marriage, relationships with families in marriage, marriage and work life, anger control, coping with violence and prevention, spending habits and its effects on marriage, effects of financial difficulties on marriage, sexual life and problems in marriage they have done.

## Conclusion and suggestions

Considering the negativities in the relations of the spouses and intra-familial communication during the pandemic process, to protect the health of the individual, therefore the family and the society; It is of great importance to organize marriage trainings on how spouses will be a model for each other and how they will control and regulate their behaviors in this process (Ponzetti, 2016). It can be said that adding the right communication method, problem solving skills, sexuality, choosing the right spouse, and being a parent to the training programs that the participants need will play a fundamental role in marital harmony and thus in maintaining a healthy marriage.

### Suggestions

- It is obvious that marriage education is of great importance in an age when divorce is a global problem not only in the TRNC but all over the world. Using the results of this study, it is recommended to develop a marriage education program or set an example for similar studies.
- The results to be obtained by conducting both qualitative and quantitative research with different participants on the same subject can be compared with the results of this research.
- Considering the findings obtained from this study, marriage education programs to be prepared in the TRNC can be organized by the Ministry of National Education and Culture for parents in schools in the context of adult education in non-formal education format.
- In addition, the training programs to be prepared can be given in the form of public education by municipalities and non-governmental organizations (trade unions, associations and political parties) in the context of public education.

## Author contributions

NS: interview and writing. KB: supervision and interview. YS: analysis and final writing. All authors contributed to the article and approved the submitted version.

## Conflict of interest

The authors declare that the research was conducted in the absence of any commercial or financial relationships that could be construed as a potential conflict of interest.

## Publisher's note

All claims expressed in this article are solely those of the authors and do not necessarily represent those of their affiliated organizations, or those of the publisher, the editors and the reviewers. Any product that may be evaluated in this article, or claim that may be made by its manufacturer, is not guaranteed or endorsed by the publisher.

## References

[B1] Akoglu G. Karaaslan B. T. (2020). Possible psychosocial effects of COVID 19 and the isolation process on children. J. Izmir Katip Celebi Univ. Faculty Health Sci. 5, 99–103.

[B2] Alaçam S. (2020). Investigation of the relationships betweenmarital satisfaction, tendency to cheat and personality traits in married individuals (Master's thesis). Isik University, Istanbul, Turkey.

[B3] Avci O. H. (2014). Topics that university students have problems in pre-marriage relationships and want to study. Aegean J. Educ. 15, 279–299. 10.12984/eed.68094

[B4] Baştürk S. Taütepe M. (2013). Scientific Research Methods. Vize Yayıncılık.

[B5] Bilen M. (1983). Healthy Human Relationships in Family / Institutions / Society, 2rd Edn, Ankara: Memoir Publishing.

[B6] Bruine de Bruin W. Parker A. M. Strough J. (2020). Age differences in reported social networks and well-being. Psychol. Aging 35, 159–168. 10.1037/pag0000415 31697096 PMC7122684

[B7] Büyüköztürk Ş. (2011). Manual of Data Analysis for Social Sciences. Pegem Akademi.

[B8] Calvano C. Engelke L. Di Bella J. Kindermann J. Renneberg B. Winter S. M. (2021). Families in the COVID-19 pandemic: parental stress, parent mental health and the occurrence of adverse childhood experiences-results of a representative survey in Germany. Eur. Child. Adolesc. Psychiatry 1–13. 10.1007/s00787-021-01739-0 33646416 PMC7917379

[B9] Canel A. N. (2012). Marriage and Family Life. Turkey Ministry of Family and Social Policies Project, 2nd Edn. Istanbul.

[B10] Chalmers J. H. (2020). Online marriage education during COVID-19 home lockdown: a multiple-baseline single-case experimental design. Interpersona 14, 150–168. 10.5964/ijpr.v14i2.3971

[B11] Creswell J. D. (2017). Mindfulness interventions. Ann. Rev. Psychol. 68, 491–516. 10.1146/annurev-psych-042716-051139 27687118

[B12] DeMaria R. M. (2005). Distressed couples and marriage education. Fam. Relat. 54, 242–253. 10.1111/j.0197-6664.2005.00019.x

[B13] Duncan S. F. Box G. ve Silliman B. (1996). Racial and gender effects on perceptions of marriage preparation programs among college-educated young adults. Fam. Relat. 45, 80–90. 10.2307/584773

[B14] Erlandson D. A. Harris E. L. Skipper B. L. Allen S. T. (1993). Doing Naturalistic Inquiry: A Guide to Methods. Sage: Beverly Hills, CA.

[B15] Evran S. (2018). The effects of traditional relative relationships on new marriages: the case of afyonkarahisar province (Master's thesis). Afyon Kocatepe University Institute of Social Sciences, Afyonkarahisar, Turkey.

[B16] Fritsch I. (1985). Parents Divorcing. Istanbul: Afa Publications

[B17] Guetto R. Vignoli D. Bazzani G. (2021). Marriage and cohabitation under uncertainty: the role of narratives of the future during the COVID-19 pandemic. Eur. Soc. 23, S674–S688. 10.1080/14616696.2020.1833359

[B18] Guner K. Z. (2014). The Relationship Between Early Maladaptive Schemas, Cognitive Processes in Relationships, Attributions, Ways of Coping With Marital Problems and Marital Adjustment (Master's Thesis). Istanbul: Maltepe University Institute of Social Sciences.

[B19] Gur B. S. Kurt T. (2011). Educational needs of families in Turkey. J. Fam. Soc. 12, 27.

[B20] Gurel B. (2016). The Effect of Economic Factors on Marriage Quality: A Dyadic Model Test. (Doctoral Thesis) Ankara: Hacettepe University.

[B21] Hamamci Z. Buga A. Duran S. (2011). Examination of information resources of university students regarding marriage experience and pre-marriage education needs. Fam. Soc. 7, 35.

[B22] Harland T. (2014). Learning about case study methodology to research higher education. High. Educ. Res. Dev. 33, 1113–1122, 10.1080/07294360.2014.911253

[B23] Hawkins A. J. Fackrell T. A. (2010). Does relationship and marriage education for lower-income couples work? A meta-analytic study of emerging research. J. Couple Relatsh. Ther. 9, 181–191. 10.1080/15332691003694927

[B24] Holland J. A. De Valk H. A. (2013). Ideal ages for family formation among immigrants in Europe. Adv. Life Course Res. 18, 257–269. 10.1016/j.alcr.2013.08.002 24796710

[B25] Hosgor E. (2013). Examination of married couples' anger expression styles and marital adjustment (PhD thesis). DEU Institute of Educational Sciences, Izmir, Turkey.

[B26] Hughes R. Bowers J. R. Mitchell E. T. Curtiss S. Ebata A. T. (2012). Developing online family life prevention and education programs. Fam. Relat. 61, 711–727. 10.1111/j.1741-3729.2012.00737.x

[B27] Hunt O. (2007). A Mixed Method Design. Article Valley. Available online at: http://www.articlealley.com/ (accessed October 27, 2022).

[B28] Kanter J. B. Williams D. T. Rauer A. J. (2021). Strengthening lower-income families: lessons learned from policy responses to the COVID-19 pandemic. Fam. Process 60, 1389–1402. 10.1111/famp.12716 34553388 PMC8652884

[B29] Kashirskaya I. K. Zholudeva S. V. Skrynnik N. E. (2015). Psychological readiness for marriage as personal formation. Mediterr. J. Soc. Sci. 6, 452. 10.5901/mjss.2015.v6n6s1p452

[B30] Lee J. Chin M. Sung M. (2020). How has COVID-19 changed family life and wellbeing in Korea? J. Comp. Fam. Stud. 51, 301–313. 10.3138/jcfs.51.3-4.006

[B31] Mahase E. (2020). Covid-19: EU states report 60% increase in domestic violence emergency calls. BMJ 369, m1872.m1872 32393463 10.1136/bmj.m1872

[B32] Martin P. D. Specter G. Martin M. ve Martin D. (2003). Expressed attitudes of adolescents toward marriage and family life. Adolescence 38, 359–367. 14560887

[B33] Mehrolhassani M. H. Yazdi-Feyzabadi V. Rajizadeh A. (2018). Evaluation of pre-marriage counseling program in Iran: a narrative review of structural, procedural, and outcome dimensions. Evid. Based Health Policy Manag. Econ. 2, 208–225.

[B34] Nekoolaltak M. Keshavarz Z. Simbar M. Nazari A. M. Baghestani A. R. (2019). Sexual compatibility among Iranian couples: a qualitative study. Sex. Relatsh. Ther. 34, 23–39. 10.1080/14681994.2017.1347615

[B35] Özdemir M. (2010). Qualitative data analysis: a study of methodology in social sciences. Eskişehir Osmangazi Univ. Soc. Sci. J. 11, 323–343.

[B36] Öztürk C. S. Arkar H. (2014). Relationships between marital adjustment and sexual satisfaction in married couples. Lit. Sympos. 1, 16–24.

[B37] Ponzetti J. J. Jr (2016). Evidence-Based Approaches to Relationship and Marriage Education. New York, NY: Routledge Taylor & Francis.

[B38] Salman N. Karaaziz M. Keskindag B. (2016). Examining relationships between marital adjustment, tendency for conflict and cheating among married couples. J. Int. Scient. Res. 3, 377–388. 10.21733/ibad.397643

[B39] Sarica K. (2018). The perception of violence against women by university students in the TRNC (Master Thesis). Institute of Health Sciences, Ankara University, Ankara, Turkey.

[B40] Silliman B. Schumm W. R. ve Jurich A. (1992). Young adults' preferences for premarital preparation program designs: An exploratory study. Contemp. Family Ther. 14, 89–100.

[B41] Silliman B. ve Schumn W. R. (2004). Adolescent perceptions of marriage and premarital couples education. Family Relat. 53, 513–520. 10.1111/j.0197-6664.2004.00060.x

[B42] Singletary B. Schmeer K. K. Purtell K. M. Sayers R. C. Justice L. M. Lin T. J.. (2022). Understanding family life during the COVID-19 shutdown. Fam. Relat. 71, 475–493. 10.1111/fare.12655 35600938 PMC9111772

[B43] Snyder I. B. Duncan S. F. Larson J. H. (2010). To assess perceived marriage education needs and interests among Latinos in a selected western community. J. Comp. Fam. Stud. 41, 347–367. 10.3138/jcfs.41.3.347

[B44] Starks H. Trinidad S. B. (2007). Choose your method: A comparison of phenomenology, discourse analysis, and grounded theory. Qual. Health Res. 17, 1372–1380. 10.1177/1049732307307031 18000076

[B45] Sürerbiçer F. S. (2008). Marriage Education Requirement According to Divorced Individuals' Experiences (Master Thesis) Ankara: Ankara University.

[B46] Taubman-Ben-Ari O. Ben-Yaakov O. ve Chasson M. (2021). Parenting stress among new parents before and during the COVID-19 pandemic. Child Abuse Negl. 117, 105080. 10.1016/j.chiabu.2021.105080 33930664 PMC9754858

[B47] TUBA (Turkish Academy of Sciences) (2020). COVID 19 Pandemic Assessment Report. Available online at: http://www.tuba.gov.tr/files/images/2020/kovidraporu/Covid-19%20Raporu-Final+.pdf (accessed January 12, 2021).

[B48] Yildirim A. Simşek H. (2011). Qualitative Research Methods in Social Sciences, 8 th Ed Ankara: Seçkin Publishing.

[B49] Yusof S. N. M. Abd Hamid N. H. Sukor N. M. (2017). Investigating the needs to develop an integrated holistic marriage education module (IHMEM). Int. J. Acad. Res. Bus. Soc. Sci. 7, 156–165. 10.6007/IJARBSS/v7-i13/3191

[B50] Zöhre K. Kocabıyık O. Bacıoğlu S. D. (2021). Marriage, and marital problems and solutions from the perspective of couples in arranged marriage. OPUS J Soc. Res. 18, 5187–5207. 10.26466/opus.898866

